# Estimating the Bed-Load Layer Thickness in Open Channels by Tsallis Entropy

**DOI:** 10.3390/e21020123

**Published:** 2019-01-29

**Authors:** Zhongfan Zhu, Jingshan Yu

**Affiliations:** Beijing Key Laboratory of Urban Hydrological Cycle and Sponge City Technology, College of Water Sciences, Beijing Normal University, Beijing 100875, China

**Keywords:** Tsallis entropy, probability distribution, bed-load, thickness, open channels

## Abstract

In the research field of river dynamics, the thickness of bed-load is an important parameter in determining sediment discharge in open channels. Some studies have estimated the bed-load thickness from theoretical and/or experimental perspectives. This study attempts to propose the mathematical formula for the bed-load thickness by using the Tsallis entropy theory. Assuming the bed-load thickness is a random variable and using the method for the maximization of the entropy function, the present study derives an explicit expression for the thickness of the bed-load layer as a function with non-dimensional shear stress, by adopting a hypothesis regarding the cumulative distribution function of the bed-load thickness. This expression is verified against six experimental datasets and are also compared with existing deterministic models and the Shannon entropy-based expression. It has been found that there is good agreement between the derived expression and the experimental data, and the derived expression has a better fitting accuracy than some existing deterministic models. It has been also found that the derived Tsallis entropy-based expression has a comparable prediction ability for experimental data to the Shannon entropy-based expression. Finally, the impacts of the mass density of the particle and particle diameter on the bed-load thickness in open channels are also discussed based on this derived expression.

## 1. Introduction

**Figure 1:**
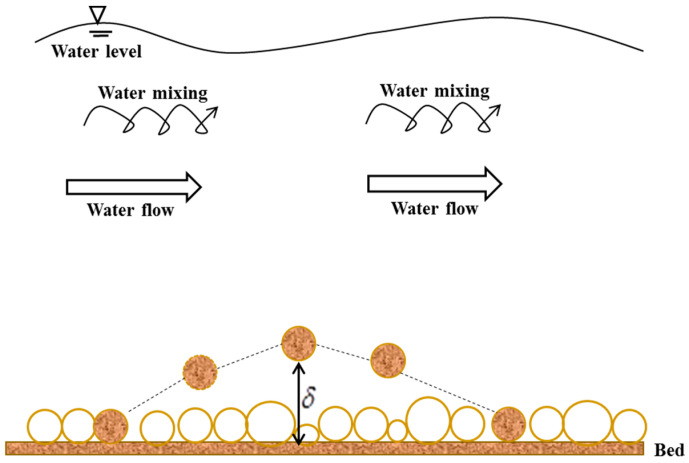
A diagram of the thickness of the bed load layer in open channel; *δ* denotes this thickness (the height of the saltation of a sediment particle near the bed).

In the context of river dynamics, the investigation of the physical mechanism of non-cohesive sediment transport in open channels is a fundamental subject that has gained great attention from researchers (e.g., [1,2,3]). There are two different sediment movement forms: bed load and suspended load. Bed-load refers to sediment particles that roll or slide on the bed or jump from the bed but rapidly drop back to the bed within a short distance [4]. The transport of bed-load is achieved via the main flow of the turbulent flow. In contrast, suspended load refers to those small-sized sediment particles that are suspended in the flow and are transported from the upstream of the river to the downstream [4]. The transport of suspended load is achieved by the fluctuating part of the turbulent flow. For bed-load discharge and suspended load discharge, completely different formulae have been developed for estimation [4,5]. However, it could be vital to determine suspended load and bed-load before estimating the total sediment discharge [4,5]. A common method is to estimate the bed-load thickness (e.g., [1,2,4,5]), defined by the saltation height of a particle, as schematically shown in Figure 1. Above this thickness, sediment transport could be estimated as suspended load, whereas sediment transport could be evaluated as bed-load below this thickness [4,5].

Some studies have examined the bed-load thickness in a sediment-laden flow by virtue of theoretically study and/or laboratory observation technique. Einstein [6] defined bed-load thickness as two grain diameters. Van Rijin [7] presented a mathematical expression of bed-load thickness, and it was related to the diameter of the particle and the capacity of flow transport. Wilson [8] put forward an analytical model for thickness as a function of dimensionless shear stress. Furthermore, by considering hydrodynamic diffusion effect due to the interactions among particles, Cheng [1] proposed an expression of the bed-load thickness, which depends on the dimensional diameter of the particle and non-dimensional shear stress. Ghoshal and Pal [2] theoretically derived an expression of the bed-load thickness as a function of the viscous coefficient of the sediment-laden flow, impact coefficient, diameter of particle, sediment density, maximum particle concentration and non-dimensional shear stress. Additionally, experimental observation and numerical works have been adopted to investigate the process of the saltation of single and/or multiple sediment particles on the bed (e.g., [9,10,11]). Further introduction into the developed formulae for the movement of the bed load layer can be found in the paper of Ghoshal and Pal [2].

As Singh et al. [12] showed, probabilistic methods by using the entropy theory have been extensively adopted to study some hydraulic problems. For example, entropy-based methods have been adopted to study the one-dimensional and two-dimensional velocity distribution of open channels (e.g., [13,14,15,16,17]), suspended sediment concentration [18,19,20], shear stress distribution [21,22], and sediment flocculation [23]. In these works, the authors showed that the entropy-based method could agree with experimental data well, indicating that the probability method by using the entropy theory could be a good addition to some existing deterministic models for tackling hydraulic engineering problems. 

Recently, Kumbhakar et al. [3] adopted the Shannon entropy concept to estimate the bed-load thickness in open channels, which motivates us to investigate the possibility of another more general form of entropy, Tsallis entropy, for evaluating the thickness of the bed-load layer. Therefore, this study attempts to propose an entropy-based formula for the bed-load layer thickness in open channels by adopting Tsallis entropy. The derived expression for the bed-load thickness is verified against collected experimental data. Furthermore, a comparison among the derived expression and other developed deterministic expressions and the Shannon entropy-based expression is also presented. 

## 2. Methodology for Determination of the Bed-Load Thickness

In Figure 1, *δ* denotes the bed-load thickness (i.e., the height of the saltation of a particle). Assuming the dimensionless bed-load layer thickness, $\bar{\delta }$, defined by *δ*/*d*, where *d* is the particle diameter, to be a random variable, this study attempts to derive the mathematical formula for the thickness of the bed-load layer based on the Tsallis entropy theory. The Tsallis entropy theory to determine the bed-load layer thickness entails the procedures as follows: (1) definition of Tsallis entropy function, (2) constraint conditions, (3) maximization of the entropy function, (4) estimation of two Lagrange multipliers, (5) hypothesis of the cumulative distribution function, and (6) estimation of a bed-load thickness expression. 

### 2.1. Definition of the Tsallis Entropy

Assume the dimensionless bed-load thickness $\bar{\delta }$ be a continuous random variable, and its probability density function is $f\bar{(\delta })$. We can express the Tsallis entropy function of the dimensionless bed-load thickness $H\bar{(\delta })$ as [24,25]:

(1) $$H\left(\bar{\delta }\right)=\frac{1}{m-1}\left\{1-\int_{{\bar{\delta }}_{\min}}^{{\bar{\delta }}_{\max}}{\left[f\left(\bar{\delta }\right)\right]}^{m}d\bar{\delta }\right\}$$

where ${\bar{\delta }}_{\min}$ and ${\bar{\delta }}_{\max}$ are the lowest and largest bounds of $\bar{\delta }$, and *m* is the parameter for the Tsallis entropy and is a real number not equal to 1. The expression $f\bar{(\delta })d\bar{\delta }$ represents the probability of dimensionless bed-load thickness occurring between $\bar{\delta }$, and $\bar{\delta }$ + $\bar{\delta }$.

### 2.2. Constraint Equations

The constraint equations are:

(2) $$\int_{{\bar{\delta }}_{\min}}^{{\bar{\delta }}_{\max}}f\left(\bar{\delta }\right)d\bar{\delta }=1$$

(3) $$\int_{{\bar{\delta }}_{\min}}^{{\bar{\delta }}_{\max}}\bar{\delta }f\left(\bar{\delta }\right)d\bar{\delta }={\bar{\delta }}_{m}$$

where ${\bar{\delta }}_{m}$ is the mean value of $\bar{\delta }$. Equation (3) is the mean constraint. 

### 2.3. Maximization of Entropy

The maximum entropy principle developed by Jaynes [26,27,28] was adopted in this study to determine among those probability density functions in accordance with the constraint conditions: Equations (2) and (3). Thus, we adopted the Euler-Lagrange calculus technique [12]. The Lagrangian function *L* is expressed as:

(4) $$\begin{array}{l} L=\frac{1}{m-1}\left\{1-\int_{{\bar{\delta }}_{\min}}^{{\bar{\delta }}_{\max}}{\left[f\left(\bar{\delta }\right)\right]}^{m}d\bar{\delta }\right\}-\lambda_{0}\left[\int_{{\bar{\delta }}_{\min}}^{{\bar{\delta }}_{\max}}f\left(\bar{\delta }\right)d\bar{\delta }-1\right]\\ -\lambda_{1}\left[\int_{{\bar{\delta }}_{\min}}^{{\bar{\delta }}_{\max}}\bar{\delta }f\left(\bar{\delta }\right)d\bar{\delta }-{\bar{\delta }}_{m}\right] \end{array}$$

where *λ*_0_ and *λ*_1_ are the Lagrange multipliers. Taking the derivative of Equation (4) with respect to $f\left(\bar{\delta }\right)$ and letting it equal to zero could yield:

(5) $$\frac{\partial L}{\partial f}=0\Rightarrow \frac{1}{m-1}\left\{1-m{\left[f\left(\bar{\delta }\right)\right]}^{m-1}\right\}-\lambda_{0}-\lambda_{1}\bar{\delta }=0$$

which results in the following expression for $f\left(\bar{\delta }\right)$:

(6) $$f\left(\bar{\delta }\right)={\left[\frac{m-1}{m}\left(\frac{1}{m-1}-\lambda_{0}-\lambda_{1}\bar{\delta }\right)\right]}^{\frac{1}{m-1}}$$

Therefore, the cumulative distribution function (CDF) of $\bar{\delta }$ could be calculated by its integration from ${\bar{\delta }}_{\min}$ to $\bar{\delta }$ as follows:

(7) $$\begin{array}{l} F\left(\bar{\delta }\right)=\int_{{\bar{\delta }}_{\min}}^{\bar{\delta }}f\left(\bar{\delta }\right)d\bar{\delta }\\ ={\left(\frac{m-1}{m}\right)}^{\frac{m}{m-1}}\frac{1}{\lambda_{1}}\left[{\left(\frac{1}{m-1}-\lambda_{0}-\lambda_{1}{\bar{\delta }}_{\min}\right)}^{\frac{m}{m-1}}-{\left(\frac{1}{m-1}-\lambda_{0}-\lambda_{1}\bar{\delta }\right)}^{\frac{m}{m-1}}\right] \end{array}$$

Substituting Equation (6) into Equation (1) could yield the maximum entropy function $H(\bar{\delta }$) as follows:

(8) $$H\left(\bar{\delta }\right)=\frac{1}{m-1}\left\{\begin{array}{l} \left({\bar{\delta }}_{\max}-{\bar{\delta }}_{\min}\right)+{\left(\frac{m-1}{m}\right)}^{\frac{m}{m-1}}\frac{1}{\left(2m-1\right)}\frac{1}{\lambda_{1}}\\ {}*\left[{\left(\frac{1}{m-1}-\lambda_{0}-\lambda_{1}{\bar{\delta }}_{\max}\right)}^{\frac{2m-1}{m-1}}-{\left(\frac{1}{m-1}-\lambda_{0}-\lambda_{1}{\bar{\delta }}_{\min}\right)}^{\frac{2m-1}{m-1}}\right] \end{array}\right\}$$

The two Lagrange multipliers, *λ*_0_ and *λ*_1_ could be determined as described below.

### 2.4. Estimation of Lagrange Multipliers

The two Lagrange multipliers, *λ*_0_ and *λ*_1_, could be estimated by substituting Equation (6) into the constraint equations. Inserting Equation (6) into Equation (2) could lead to:

(9) $$\frac{1}{\lambda_{1}}{\left(\frac{m-1}{m}\right)}^{\frac{m}{m-1}}\left[{\left(\frac{1}{m-1}-\lambda_{0}-\lambda_{1}{\bar{\delta }}_{\min}\right)}^{\frac{m}{m-1}}-{\left(\frac{1}{m-1}-\lambda_{0}-\lambda_{1}{\bar{\delta }}_{\max}\right)}^{\frac{m}{m-1}}\right]=1$$

Inserting Equation (6) in Equation (3) one could get:

(10) $$\begin{array}{l} {\bar{\delta }}_{\max}{\left(\frac{1}{m-1}-\lambda_{0}-\lambda_{1}{\bar{\delta }}_{\max}\right)}^{\frac{m}{m-1}}-{\bar{\delta }}_{\min}{\left(\frac{1}{m-1}-\lambda_{0}-\lambda_{1}{\bar{\delta }}_{\min}\right)}^{\frac{m}{m-1}}\\ +\frac{m-1}{2m-1}\frac{1}{\lambda_{1}}\left[{\left(\frac{1}{m-1}-\lambda_{0}-\lambda_{1}{\bar{\delta }}_{\max}\right)}^{\frac{2m-1}{m-1}}-{\left(\frac{1}{m-1}-\lambda_{0}-\lambda_{1}{\bar{\delta }}_{\min}\right)}^{\frac{2m-1}{m-1}}\right]\\ +\lambda_{1}{\bar{\delta }}_{m}{\left(\frac{m}{m-1}\right)}^{\frac{m}{m-1}}=0 \end{array}$$

These two equations can be solved numerically when the values of ${\bar{\delta }}_{\min}$, ${\bar{\delta }}_{\max}$, ${\bar{\delta }}_{m}$ and *m* are known.

### 2.5. Hypothesis on Cumulative Distribution Function

In order to propose the mathematical expression for the dimensionless bed-load thickness in the real (space) domain, a hypothesis regarding the CDF of the dimensionless bed-load thickness needs to be made [12].

Previous studies have indicated that the thickness of the bed-load movement is largely related to the dimensionless shear stress $\tau_{*}$ [1,2,29,30]. Recently, Kumbhakar et al. [3] put forward the following power-type hypothesis regrading CDF to be a good choice:

(11) $$F(\bar{\delta })={\left(\frac{\tau_{*}-\tau_{*\min}}{\tau_{*\max}-\tau_{*\min}}\right)}^{\eta }$$

where $\tau_{*\max}$ and $\tau_{*\min}$ are upper and lower values of $\tau_{*}$, and *η* is a real parameter.

### 2.6. Estimation of an Expression for Bed-Load Layer Thickness

By combining Equations (7), (9) and (11), the dimensionless thickness of the bed-load layer $\bar{\delta }$ could be expressed as:

(12) $$\begin{array}{l} \bar{\delta }=-\frac{1}{\lambda_{1}}{\left\{\begin{array}{l} {\left(\frac{1}{m-1}-\lambda_{0}-\lambda_{1}{\bar{\delta }}_{\min}\right)}^{\frac{m}{m-1}}-\\ \left[{\left(\frac{1}{m-1}-\lambda_{0}-\lambda_{1}{\bar{\delta }}_{\min}\right)}^{\frac{m}{m-1}}-{\left(\frac{1}{m-1}-\lambda_{0}-\lambda_{1}{\bar{\delta }}_{\max}\right)}^{\frac{m}{m-1}}\right]{\left(\frac{\tau_{*}-\tau_{*\min}}{\tau_{*\max}-\tau_{*\min}}\right)}^{\eta } \end{array}\right\}}^{\frac{m-1}{m}}\\ -\frac{\lambda_{0}}{\lambda_{1}}+\frac{1}{\lambda_{1}\left(m-1\right)} \end{array}$$

Equation (12) represents the Tsallis entropy-based expression for the bed-load layer thickness in open channels.

## 3. Comparison with Laboratory Data Sets and Discussion

### 3.1. Selection of Laboratory Data Sets

Six laboratory data sets collected from the literature are adopted in this study to check the accuracy of the expression derived by the Tsallis entropy (Equation (12)). These include Sekine and Kikkawa [31], a smooth bed studied by Hu and Hui [29], a rough bed studied by Hu and Hui [30], Sumer et al. [32], Lee et al. [33] and Bhattacharyya et al. [34]. The laboratory data sets of the thickness of the bed-load layer are fairly limited, possibly because there could be measurement limitations in tracking the entire process of the saltation of the particles on the bed in the experiment [1]. When there are few sediment particles on the bed, a camera imaging system could be adopted to track the saltation behaviour of a particle on the bed in the laboratory, as shown in collected experimental data sets [29,30,31,32,33,34]; however, for many sediment particles moving on the bed, some typical bed forms (such as ripples and dunes) will be formed, for which there seems to lack some effective measurement techniques to track particle movement [1]. The hydraulic conditions for six collected experimental data sets is summarized in Table 1, and the collected data include the particle with different materials (sediment or plastic), diverse particle size, and various shear stress conditions (a low shear stress environment, for example, $\tau_{*}$ = 0.04, 0.05, in the work of Lee et al. [33] and Sekine and Kikkawa [31]; a strong shear condition, for example, $\tau_{*}$ = 2.50 in the work of Sumer et al. [32]). In the third column of this table, *s* is the specific gravity of the particle, and it is the mass density of the particle divided by the density of the fluid. In the fourth column, $d_{*}$ is the dimensionless diameter of the particle, given by the expression d∗=[(s−1)gνf2]1/3 adopted by some studies [1,2,33], where *g* is the acceleration of the gravity, and $\nu_{f}$ is the kinematic viscosity of fluid. In the last column, $\tau_{*}$ is the dimensionless shear stress as mentioned above, and is calculated as τ∗=u∗2(s−1)gd, where $u_{*}$ is the shear velocity [1,29,30].

An error analysis is used to determine the accuracy of the developed expressions with collected laboratory data sets by calculating the average value of the relative error *R* in percent as:

(13) $$\frac{100}{N}\left[\sum_{i=1}^{N}\left|\frac{m_{i}-o_{i}}{o_{i}}\right|\right]$$

in which *m* and *o* are the estimated and observed points, respectively, and *N* is the total number of data points. The fitting effect increases when *R* decreases. 

### 3.2. Some Developed Deterministic Models

There have been many studies to predict the bed-load thickness from different perspectives. Van Rijin [7] proposed that the saltation height *δ* of a sediment particle could be computed as:

(14) $$\frac{\delta }{d}=0.3d_{*}^{0.7}T_{*}^{0.5}$$

where $d_{*}$ is the non-dimensional diameter of the particle, as already mentioned, and $T_{*}$ is the non-dimensional capacity of flow transport given by T∗=τ∗−τ∗cτ∗c, where $\tau_{*c}$ is the dimensionless critical shear stress. Wilson [8] analytically derived the expression of *δ* as:

(15) $$\frac{\delta }{d}=10\tau_{*}$$

From the mechanical and stochastic perspectives, Hu and Hui [29,30] put forward the expressions of *δ* for smooth and rough beds respectively as follows:

(16) $$\frac{\delta }{d}=3.65s^{1.05}\tau_{*}^{0.82},\;\mathrm{for}\;\mathrm{smooth}\;\mathrm{bed}$$

(17) $$\frac{\delta }{d}=1.78s^{0.86}\tau_{*}^{0.69},\;\mathrm{for}\;\mathrm{rough}\;\mathrm{bed}$$

Similar to Equation (14), researchers have proposed different expressions of *δ*, including δd=1.112d∗0.325T∗0.511 in the work of Lee et al. [35], δd=0.301d∗0.4522T∗0.3345 in Lee et al. [33], δd=1.091d∗0.330T∗0.511 in Wang et al. [36], and δd=90d∗−0.66T∗−0.07 in the study of Kharlamova and Vlasak [37], respectively. 

By considering the hydrodynamic diffusion effect because of the interactions among particles, Cheng [1] derived an expression for the bed-load layer thickness. The derived expression of *δ*/*d* was given by:

(18) $$\frac{\delta }{d}=\tau_{*}\frac{d_{*}^{3}}{{\left(\sqrt{25+1.2d_{*}^{2}}-5\right)}^{1.5}}\left(\int_{0}^{c_{b}}\frac{E_{*}}{c\mu_{r}\omega_{r}}dc\right)$$

In this expression, $E_{*}$ is the non-dimensional diffusion coefficient, *c* is the volumetric concentration of the particle, *c_b_* is the maximum bed concentration, *μ_r_* is the dynamic viscosity of the sediment-fluid mixture divided by the fluid viscosity, and *ω_r_* is the settling velocity of a particle in the mixture divided by that in the clear fluid. The parameters $E_{*},$ *μ_r_* and *ω_r_* were given by:

(19) $$E_{*}=a_{i}{\left(c^{-\frac{1}{3}}-1\right)}^{-2}$$

(20) $$\mu_{r}=\exp\left\{\frac{2.5}{\beta }\left[{\left(1-c\right)}^{-\beta }-1\right]\right\}$$

(21) $$\omega_{r}=\frac{\mu_{r}}{1+\Delta *c}{\left(\frac{\sqrt{25+1.2d_{*}^{2}{\left(1-c\right)}^{\frac{2}{3}}{\left(1+\Delta *c\right)}^{\frac{2}{3}}\mu_{r}^{-\frac{4}{3}}}-5}{\sqrt{25+1.2d_{*}^{2}}-5}\right)}^{1.5}$$

where Δ=s−1, *a_i_* is the impact coefficient and is taken to be 0.0041 as suggested by Ghoshal and Pal [2], and the value of *β* is taken to be 2.5 by Cheng [1]. More details regarding Equation (18) are found in the work of Cheng [1]. 

By analysing the impact shear stress because of the interactions among particles and viscous shear stress because of the interaction between the particle and the surrounding fluid, Ghoshal and Pal [2] analytically derived an expression for *δ*/*d* as follows:

(22) δd=av(Re)∗s∫0cbμrc13(c−13−1)cωrdc+aiτ∗d∗3(Re)∫0cb1μr(c−13−1)2cωrdc

where Re (=*ω*_0_*d*/*ν_f_*) is the Reynolds number of sediment particles (here *ω*_0_ is the settling velocity of a particle in still and clear fluid), and *a_ν_* is the proportionality constant called the viscous coefficient. A more detailed introduction of this expression is found in the study of Ghoshal and Pal [2].

In the models of Van Rijin [7], Lee et al. [35], Lee et al. [33], Wang et al. [36], and Kharlamova and Vlasak [37], the determination of the value of $\tau_{*c}$ is required. To calculate it, we adopted the expression τ∗c=0.24d∗+0.055[1−exp(−0.02d∗)], suggested by Soulsby and Whitehouse [38].

### 3.3. Comparsion Results

**Figure 2:**
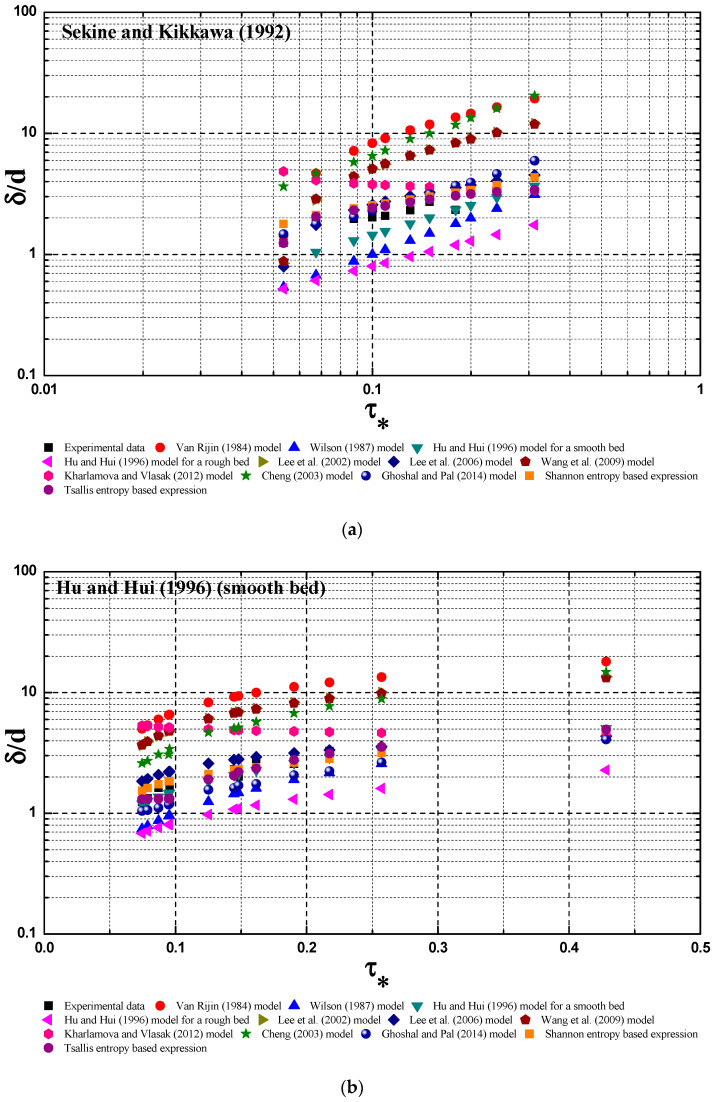

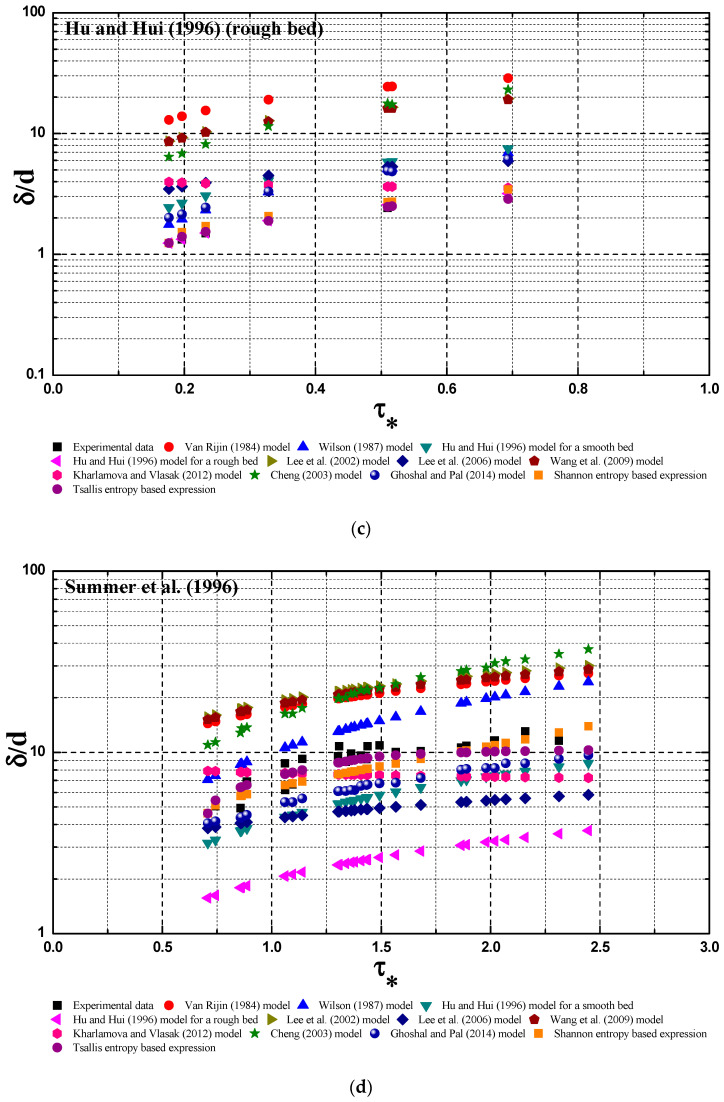

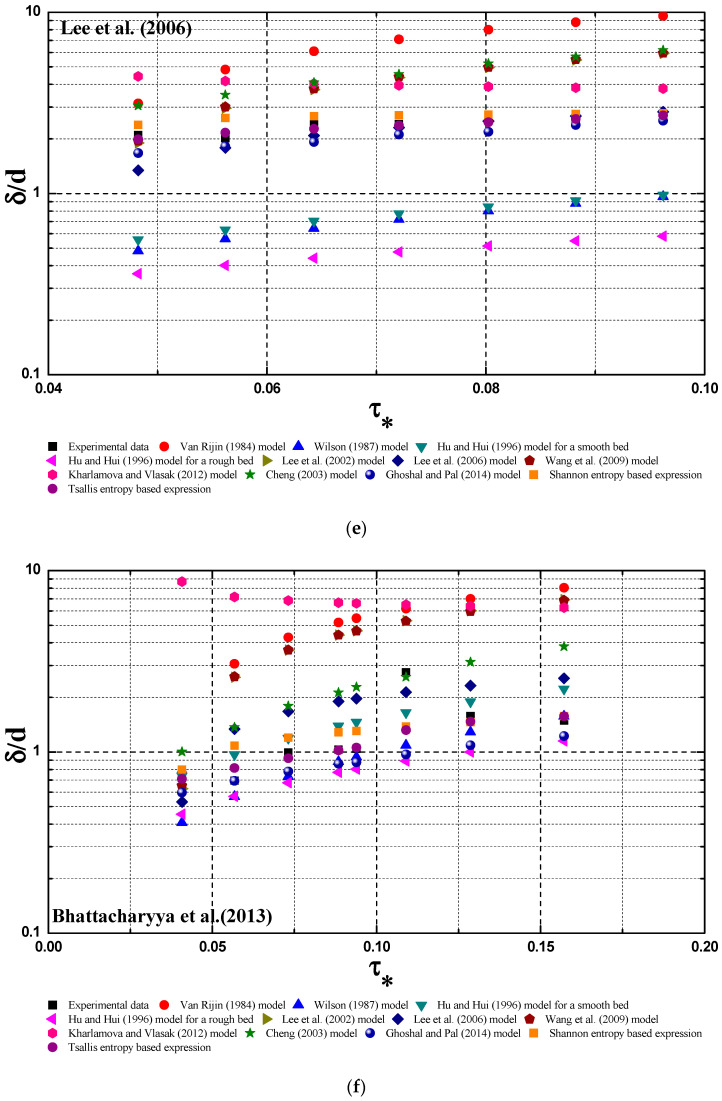
Comparison of the derived expression by the Tsallis entropy (Equation (12)), ten existing deterministic models, and the Shannon entropy-based expression with six laboratory data sets, including Sekine and Kikkawa [31] (**a**), a smooth bed in Hu and Hui [29] (**b**), a rough bed in Hu and Hui [30] (**c**), Sumer et al. [32] (**d**), Lee et al. [33] (**e**) and Bhattacharyya et al. [34] (**f**). Ten deterministic models are the Van Rijin [7] model, Wilson [8] model, Hu and Hui [29] model for a smooth bed, Hu and Hui [30] model for a rough bed, Lee et al. [35] model, Lee et al. [33] model, Wang et al. [36] model, Kharlamova and Vlasak [37] model, Cheng [1] model and Ghoshal and Pal [2] model, as mentioned above in the text.

For each laboratory data set, the two Lagrange multipliers in Equation (12), *λ*_0_ and *λ*_1_, could be estimated by solving the non-linear equation system (Equations (9) and (10)) when the values of ${\bar{\delta }}_{\min}$, ${\bar{\delta }}_{\max}$, ${\bar{\delta }}_{m}$ and *m* are known are known from the laboratory data. By fitting Equation (11) with collected laboratory data, the value for *η* could be estimated. Figure 2 shows the comparison of the proposed Tsallis entropy-based expression (Equation (12)), and ten existing deterministic models, as well as the Shannon entropy-based expression given by Kumbhakar et al. [3], with six laboratory data sets. Table 2 lists the calculated *R* values between the Tsallis entropy based expression and each data set, as well as the values of the Lagrange multipliers *λ*_0_ and *λ*_1_, and the fitting parameter *η*. Table 3 presents the comparison result (i.e., the calculated *R* value) among ten existing deterministic models, the Shannon entropy-based expression given by Kumbhakar et al. [3] and each data set, as well as the Tsallis entropy-based expression.

From the last column of Table 2, it can be seen that there is a low *R* value for each case: in particular, the *R* values fall below 6 for experimental data for Hu and Hui [30] (rough bed), Lee et al. [33], and Bhattacharyya et al. [34].This observation indicates that the Tsallis entropy expression could agree with laboratory data well, considering that there is scattering in some experimental data (for example Sumer et al. [32], and Sekine and Kikkawa [31]). 

In each column of Table 3, the symbol *** corresponds to the minimum error. As seen, the Tsallis entropy-based expression has the minimum fitting error in all real cases, except for the case of Hu and Hui [30] (rough bed). For the experimental data from Hu and Hui [30] (rough bed), the formula proposed by Hu and Hui [30] gives the best fitting result because this formula is completely derived from the data points of Hu and Hui [30] on a rough sediment bed. For the case of Hu and Hui [29] (smooth bed), the formula proposed by Hu and Hui [29] could be in good agreement with data points, since it is derived from these data (*R* = 7.33), and the Tsallis entropy-based expression could have a similarly good agreement (*R* = 7.31). Thus, this study shows that the derived expression by the Tsallis entropy could have a better prediction accuracy for the bed-load thickness of the sediment particle on the bed in the open channel than some existing deterministic models. Comparing the last two rows of Table 3, it could be found that the derived Tsallis entropy-based expression has a comparable prediction ability for experimental data to the Shannon entropy-based expression, considering some uncertainty associated with the experimental data.

### 3.4. Physical Interpretation

In the study of Kumbhakar et al. [3], the lower and upper limits of the bed-load thickness, ${\bar{\delta }}_{\min}$ and ${\bar{\delta }}_{\max}$ are computed as follows:

(23) $${\bar{\delta }}_{\min}=8.4823s^{-1.3598}d_{*}^{-0.135}$$

and:

(24) $${\bar{\delta }}_{\max}=420.36s^{-0.0534}d_{*}^{-1.0112}$$

which shows a high correlation coefficient of fitting (above 0.98) with the experimental data. Substituting Equations (23) and (24) in the Tsallis entropy-based expression (Equation (12)), and adopting *λ*_0_ = –17.90 and *λ*_1_ = 6.48 as used for the experimental data of Lee et al. [34] as a typical example, we attempt to analyse the impact of the variation of particle properties, including the specific gravity of the particle *s* and the diameter of the particle *d*, on the thickness of the bed-load layer in the open channel. 

**Figure 3:**
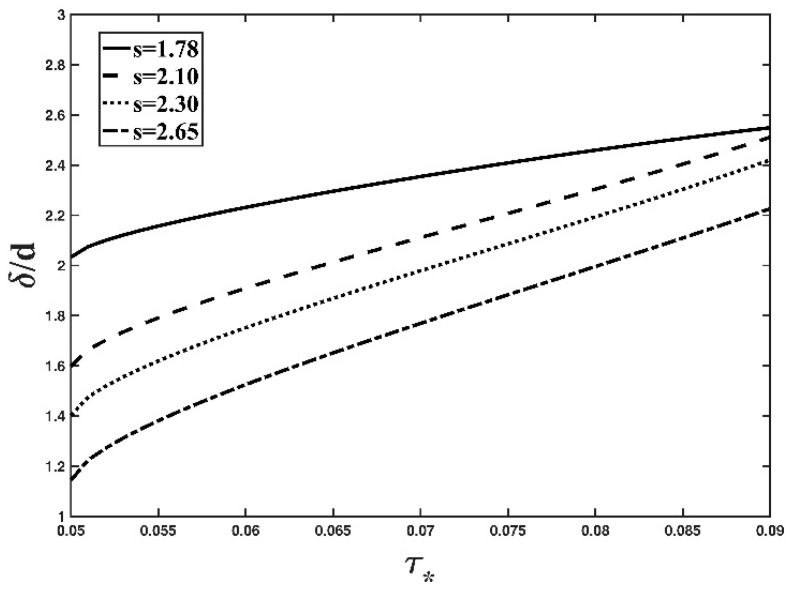
Variation in the dimensionless thickness of the bed-load layer with respect to the non-dimensional shear stress, under different values of specific gravity of the particle (*d* = 6 mm).

Fixing the value of the particle diameter, Figure 3 presents the effect of the specific gravity of the particle on the bed-load thickness based on the Tsallis entropy-based expression (Equation (12)). As observed from this figure, the bed-load thickness decreases as s increases. An increase in s leads to the increase of the particle density. Thus the gravity of the particle could increase for a fixed particle diameter. Therefore, under the same hydrodynamic condition of the flow in the open channel, the heavy particle could jump vertically lower than the light particle. Consequently, the bed-load thickness decreases. 

**Figure 4:**
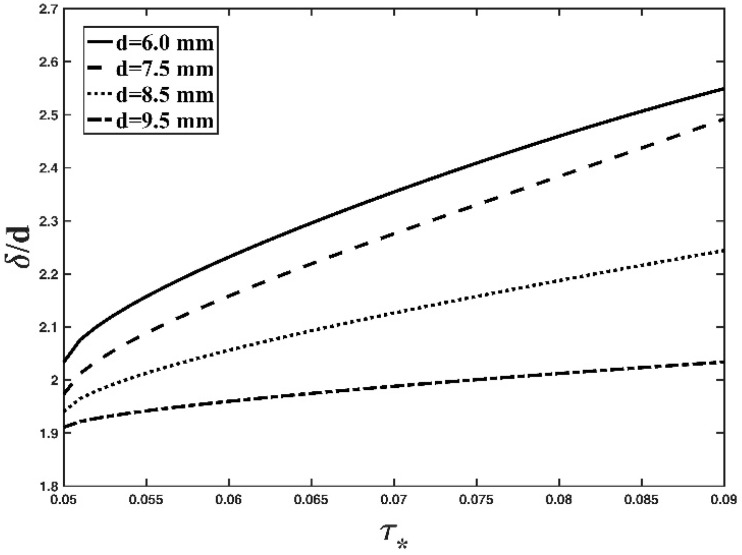
Variation in the dimensionless thickness of the bed-load layer with respect to the non-dimensional shear stress, with various particle diameter values (*s* = 1.78).

Fixing the value of the specific gravity of the particle, Figure 4 demonstrates the effect of particle diameter on the bed-load thickness based on the Tsallis entropy-based expression (Equation (12)). As seen from this figure, the thickness of the bed-load layer decreases as *d* increases. The physical explanation is similar to that mentioned above. With the increase in particle diameter, the particle gravity will increase for a fixed mass density of the particle, and the large particle could jump vertically lower than the small particle on the bed under the same hydrodynamic condition in the open channel. Consequently, the increase in particle diameter leads to the decrease of the thickness of the bed-load layer. These conclusions are in accordance with the studies of Cheng [1] and Ghoshal and Pal [2], and also with our understanding of the dynamic mechanisms of the bed load transport in an open channel.

Equation (12) could predict the bed-load thickness in the open channel with high accuracy, as shown by Figure 2, as long as the values of ${\bar{\delta }}_{\min}$, ${\bar{\delta }}_{\max}$ and ${\bar{\delta }}_{m}$ are known. The mathematical form of this expression is simpler than the existing deterministic models (for example the Cheng [1] model and Ghoshal and Pal [2] model). Equation (12) provides a new expression for estimating the bed-load thickness in an open channel based on the Tsallis entropy theory, as an addition to existing deterministic models. However, it could point out that Equation (12) contains fewer physical properties than existing deterministic models. For example, in the study of non-cohesive sediment transport, the impact among solid particles near the bed, which generates impact shear stress, and the dynamic viscosity of the mixture play a role in determining bed load transport [1,2]. In the Cheng [1] model and Ghoshal and Pal [2] model, the impact coefficient *a_i_* and relative viscosity of the mixture *μ_r_* have been adopted to characterize the impacts of impact shear stress and the viscosity of the mixture, respectively. However, the present Tsallis entropy-based expression (Equation (12)) does not incorporate these parameters.

Finally, it needs to acknowledge that the laboratory data sets regarding the bed-load thickness might have considerate uncertainty, possibly because there could be measurement uncertainty in tracking the saltation process of the particles on the bed during the experiment, as shown in collected experimental data sets [29,30,31,32,33,34]. The uncertainty in the experimental data might originate from two sources. The first could be the difficulty in tracking the particle movement near the bed, possibly due to the interference of particle crowding. The second might be the measurement error by the photographic technique. It also needs to acknowledge that the proposed entropy-based model has adopted the collected data sets when estimating the relevant coefficients of the model, similar to other works of applying entropy into some hydraulic engineering problem by some researchers [15,16,17,18,19,20,21]. Seeking a possible link between the estimated Lagrange multipliers and the boundary condition of flow structure (and/or physical property parameter of the particle) could be worthy of further investigation in future study in order to allow the model to be easily and universally used when physical property of the sediment and flow characteristic are only required.

## 4. Conclusions

This study aimed to find an expression for the thickness of the bed-load layer in an open channel. Some concluding remarks are as follows:

(1) A mathematical expression that could predict the bed-load thickness in open channel was derived by adopting the Tsallis entropy theory, coupled with the maximum entropy principle.
(2) The derived expression by the Tsallis entropy could agree with the collected six laboratory data sets fairly well.
(3) The derived expression by the Tsallis entropy was compared with other deterministic models for six collected laboratory data sets. The expression shows better prediction accuracy for experimental data than other deterministic models.
(4) The derived Tsallis entropy-based expression has a comparable prediction ability for experimental data to the Shannon entropy based expression, considering the uncertainty associated with the experimental data.
(5) Based on the Tsallis entropy based expression in this study, either an increase in mass density of the particle or in particle diameter leads to a reduction in the thickness of the bed-load layer in an open channel, in accordance with previous studies and our understanding of the dynamic mechanisms of the bed-load transport in the channel.

## Figures and Tables

**Table 1:** Information on experimental data sets collected from the literature.

Data Series	The Diameter of the Particle *d* (mm)	Specific Gravity *s*	Dimensionless Diameter of the Particle d_*_	Dimensionless Shear Stress τ_*_ Range
Sekine and Kikkawa [31]	5	2.50	122.52	0.05–0.31
Hu and Hui [29] (smooth bed)	3	2.64	75.73	0.07–0.43
Hu and Hui [30] (rough bed)	4	2.64	100.98	0.10–0.70
Sumer et al. [32]	2.6	1.14	28.90	0.70–2.50
Lee et al. [33]	6	1.78	118.23	0.04–0.10
Bhattacharyya et al. [34]	2	2.65	50.57	0.04–0.16

**Table 2:** Comparison result of the derived expression by the Tsallis entropy (Equation (12)) with collected laboratory data sets.

Data Source	Parameter Estimation				Fitting Result
	$\lambda_{0}$	$\lambda_{1}$	η	m	R
Sekine and Kikkawa [31]	−1.30	0.50	0.35	3	12.11
Hu and Hui [29] (for a smooth bed)	1.06	−0.26	1	3	7.31
Hu and Hui [30] (for a rough bed)	−2.67	1.07	0.65	3	5.26
Sumer et al. [32]	−0.18	0.06	0.5	3	8.94
Lee et al. [33]	−17.90	6.48	0.65	3	5.33
Bhattacharyya et al. [34]	−4.82	3.15	0.85	3	1.47

**Table 3:** Comparison among ten existing deterministic models, the Shannon entropy-based expression given by Kumbhakar et al. [3] and six experimental data sets from literature using calculated R value, as well as Tsallis entropy-based expression. In each column, the symbol *** corresponds to the minimum error for each case.

Experimental Data	Fitting Result: *R* Value					
	Sekine and Kikkawa [32]	Hu and Hui [29] (Smooth Bed)	Hu and Hui [30] (Rough Bed)	Sumer et al. [32]	Lee et al. [33]	Bhattacharyya et al. [34]
Van Rijin [7] model	294.17	305.29	881.50	131.04	174.93	302.16
Wilson [8] model	45.11	33.79	74.35	56.29	70.48	24.50
Hu and Hui [29] model for a smooth bed	25.32	7.33	112.73	38.39	68.32	30.73
Hu and Hui [30] model for a rough bed	60.29	50.40	2.88 ***	71.88	80.49	32.73
Lee et al. [35] model	147.78	198.67	557.56	152.62	72.19	246.15
Lee et al. [33] model	22.51	27.51	133.69	45.24	12.15	62.84
Wang et al. [36] model	147.42	197.46	548.35	142.91	73.02	244.97
Kharlamova and Vlasak [37] model	75.57	153.26	108.05	29.60	68.64	559.16
Cheng [1] model	266.08	131.53	503.15	138.30	88.09	87.75
Ghoshal and Pal [2] model	21.09	23.19	74.45	28.32	13.77	23.01
The Shannon entropy-based model	14.06	11.00	6.75	9.34	10.54	21.05
The Tsallis entropy-based model	12.11 ***	7.31 ***	5.26	8.94 ***	5.33 ***	7.59 ***
